# The Diagnostic Value of MicroRNAs as a Biomarker for Hepatocellular Carcinoma: A Meta-Analysis

**DOI:** 10.1155/2019/5179048

**Published:** 2019-11-29

**Authors:** Yao Jiang, Jimin He, Yiqin Li, Yongcan Guo, Hualin Tao

**Affiliations:** ^1^Department of Clinical Laboratory Medicine, The Affiliated Hospital of Southwest Medical University, Luzhou, China; ^2^Department of Neurosurgery, Suining Central Hospital, Suining, China; ^3^Clinical Laboratory of Traditional Chinese Medicine Hospital, Southwest Medical University, Luzhou, China

## Abstract

**Background:**

Recently, the role of microRNAs (miRNAs) in diagnosing cancer has been attracted increasing attention. However, few miRNAs have been applied in clinical practice. The purpose of this study was to evaluate the diagnostic efficacy of miRNAs for hepatocellular carcinoma (HCC) at early stages clinically.

**Methods:**

A literature search was carried out using PubMed, Web of Science, and EMBASE databases. We explored the diagnostic value of miRNAs in distinguishing HCC from healthy individuals. The quality assessment was performed in Review Manager 5.3 software. The overall sensitivity and specificity and 95% confidence intervals (CIs) were obtained with random-effects models through Stata 14.0 software. And heterogeneity was assessed using *Q* test and *I*^2^ statistics. Meta-regression and subgroup analyses were conducted based on the sample, nation, quality of studies, and miRNA profiling. The publication bias was evaluated through Deeks' funnel plot.

**Results:**

A total of 34 studies, involving in 2747 HCC patients and 2053 healthy individuals, met the inclusion criteria in the 33 included literature studies. In the summary receiver operating characteristic (sROC) curve, AUC was 0.92 (95% CI, 0.90–0.94), with 0.84 (95% CI, 0.79–0.88) sensitivity and 0.87 (95% CI, 0.83–0.90) specificity. There was no publication bias (*P*=0.48).

**Conclusion:**

miRNAs in vivo can be acted as a potential diagnostic biomarker for HCC, which can facilitate the early diagnosis of HCC in clinical practice.

## 1. Introduction

Hepatocellular carcinoma (HCC) accounts for more than 90% of primary liver cancers. It is one of the most common malignant tumors in the world and the third leading cause of cancer-related death [1], with an increasing incidence rate in the United States [2]. However, HCC is often diagnosed at an advanced stage, leading to limited treatment [3, 4] and poor prognosis, with a median overall survival of 6–20 months and 5-year survival rate of 3% [5].

HCC is often confirmed by pathological biopsy [6] and immunohistochemistry [7]. However, these methods have high invasiveness, leading to limited clinical application for early HCC screening. The alpha-fetoprotein (AFP) is the most common serum marker in the clinic for HCC routinely screening. However, it is not accurate for the diagnosis of HCC [4]. Given the cutoff value of AFP was 20 *μ*g/L [8], less than 400 *μ*g/L [9], the AFP has limited diagnostic efficacy. Besides, the imaging techniques are also usually used to early screen HCC patients, including ultrasound (US), computed tomography (CT), and magnetic resonance imaging (MRI) [10]. However, the diagnostic accuracy of the imaging techniques mainly relies on the size of the nodules, and these techniques are insensitive to small HCC nodules [10]. Because of the limited diagnostic value in the previous methods, it is urgent to discover a biomarker for the diagnosis of early HCC. Recently, many studies were focused on the role of microRNAs (miRNAs) in HCC.

miRNAs are highly conservative noncoding RNA, with 19∼25 nucleotides [11, 12], resulting in mRNA degradation or inhibiting transcript through combining with mRNA [13, 14], which play an essential role in the formation mechanism of tumor [12, 15, 16]. Moreover, it is characterized by stable in serum or plasma [17], laying a foundation for serum/plasma miRNAs in the diagnosis of tumors. In recent years, miRNAs have higher diagnostic accuracy in various cancers, such as glioma [18], prostate cancer [19], breast cancer [20], renal cell carcinoma [21], colorectal cancer [22], non-small-cell lung cancer [23], and HCC [24, 25]. When it comes to the diagnosis of HCC, Shaker et al. demonstrated that the expression levels of miR-221 and miR-101-1 could be used as noninvasive biomarkers for the diagnosis of early HCC from HCV patients [26]. Zekri et al. showed that miRNA panels might distinguish early HCC from liver cirrhosis (LC), chronic hepatitis C (CHC), and healthy individuals combining with the AFP [27]. And miR-26a was identified as a promising biomarker for the diagnosis of early HCC by Zhuang et al. [28]. However, the diagnostic efficacy of miRNAs in HCC was different. The race of participants, study design, sample type, the types of miRNAs, the size of the sample, and the background of HCC might be the main cause of inconsistent results in different studies. Therefore, it was imperative to systematically and comprehensively analyze the differences among these studies. And this article aimed at estimating the overall diagnostic efficacy of circulating miRNAs in HCC.

## 2. Materials and Methods

### 2.1. Study Search Strategies

We searched the related studies about the diagnosis of miRNAs for HCC in the Web of Science, EMBASE, and PubMed databases. There was no limit to the published time, languages, and sample source, with the deadline of searching articles (October 08, 2019). To verify the effectiveness of the study, we also manually searched the review papers and other relevant references. The search terms were found in the website of http://fmrs.metstr.com/index.aspx. “Carcinoma, hepatocellular,” “microRNAs,” and “diagnosis” were inputted in http://mesh.metstr.com/, where the entrance words were obtained for literature retrieval.

### 2.2. Study Selection

Inclusion and exclusion criteria to screen the literature were developed. A study can be included if it met the following criteria: (1) the studies were focused on the expression of miRNAs between HCC patients and healthy controls (HCs); (2) the difference in miRNA expression levels was statistically significant; (3) the data in studies must be complete, including sample size of two groups and sensitivity and specificity evaluation indexes to calculate the value of true positive (TP), false positive (FP), false negative (FN), and true negative (TN) or TP, FP, FN, and TN were directly given in the studies; (4) the purpose of studies was related to the diagnosis of HCC. In addition, the exclusion criteria were described below: (1) the studies were review, systematic evaluation, or meta-analysis; (2) the studies were duplicate; (3) the studies were focused on animal studies and cell culture, without case-control groups of humans; (4) the studies were the abstract of literature, letter to editors, or meetings; (5) the studies lacked complete information; and (6) the papers concentrated only on the survival, treatment, and prognosis of HCC, without involving in the diagnosis of HCC.

### 2.3. Quality Assessment

The qualitative evaluation of quality assessment was performed using QUADAS-2 tools for diagnostic studies, which included four domains: Patient Selection, Index Test, Reference Standard, and Flow and Timing [29, 30]. And two reviewers (Yao Jiang and Yiqin Li) performed the assessment separately. When encountering the divergence on the same literature, we invited a third individual (Jimin He) to discuss and solve the problem together.

### 2.4. Data Extraction

The data were extracted by two reviewers (Yao Jiang and Yiqin Li). The following contents need to be extracted: the first author, publication year, country, sample size, age ± standard deviation (SD), proportion of males, miRNAs categories, area under the curve (AUC), sensitivity, specificity, detection method, internal reference, and cutoff value. Besides, we pooled multiple groups of miRNAs in a single study using Meta-disc 1.4 software (https://meta-disc.software.informer.com/1.4/). Finally, the data were obtained in each study on the basis of the same specimen source, including the value of TP, FP, FN, and TN.

### 2.5. Statistical Analysis

Meta-disc 1.4 and Stata 14.0 software were used for all statistical analysis. And *P* value less than 0.05 was considered statistically significant. Pooled sensitivity and specificity statistical indicators were analyzed using a random-effects model. The overall diagnostic efficacy was evaluated by the summary receiver operating characteristic (sROC) curve. The threshold effect was investigated based on the Spearman correlation coefficient and *P* value. And the heterogeneity was assessed using *I*^2^ and chi-square test. When the value of *I*^2^ >50% and *P* value <0.05, the heterogeneity exits. The value of *I*^2^ is 0–40%, 40–70%, and 70–100%, which indicate the low, medium, and high heterogeneity, respectively [31]. In addition, meta-regression and subgroup analyses were further applied to explore the potential sources of heterogeneity. The AUC was an index of diagnostic efficacy, the values ranging from 1.0 to 0.5. The closer the AUC is to 1.0, the better the diagnostic efficacy. At last, Deeks' funnel plot was used to analyze the potential publication bias [32, 33].

## 3. Results

### 3.1. Study Selection

**Figure 1:**
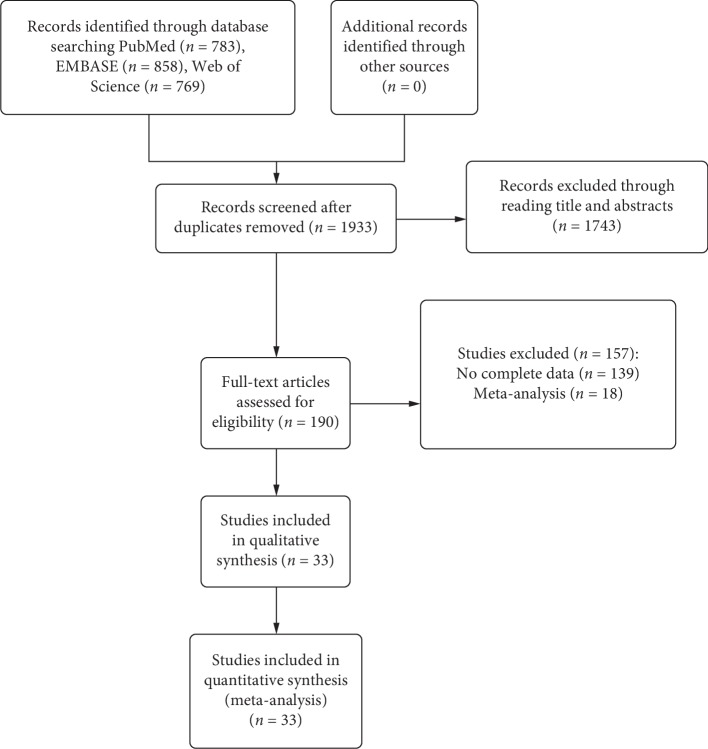
The flow diagram of study selection.

A total of 2410 related studies were found through literature retrieval, 477 of which were duplicated literature. Ultimately, 33 papers were selected for meta-analysis according to the inclusion and exclusion criteria. The flowchart of study selection is shown in Figure 1.

### 3.2. Study Characteristics

The 33 papers included 34 eligible studies, which were published between 2010 and 2019. The data involved in 2747 patients with HCC and 2053 HCs. All the studies were published in English. The characteristics of the included studies are shown in Table 1. And the diagnostic efficacy of miRNAs for HCC in the included studies is shown in [Supplementary-material supplementary-material-1].

### 3.3. Quality Assessment

**Figure 2:**
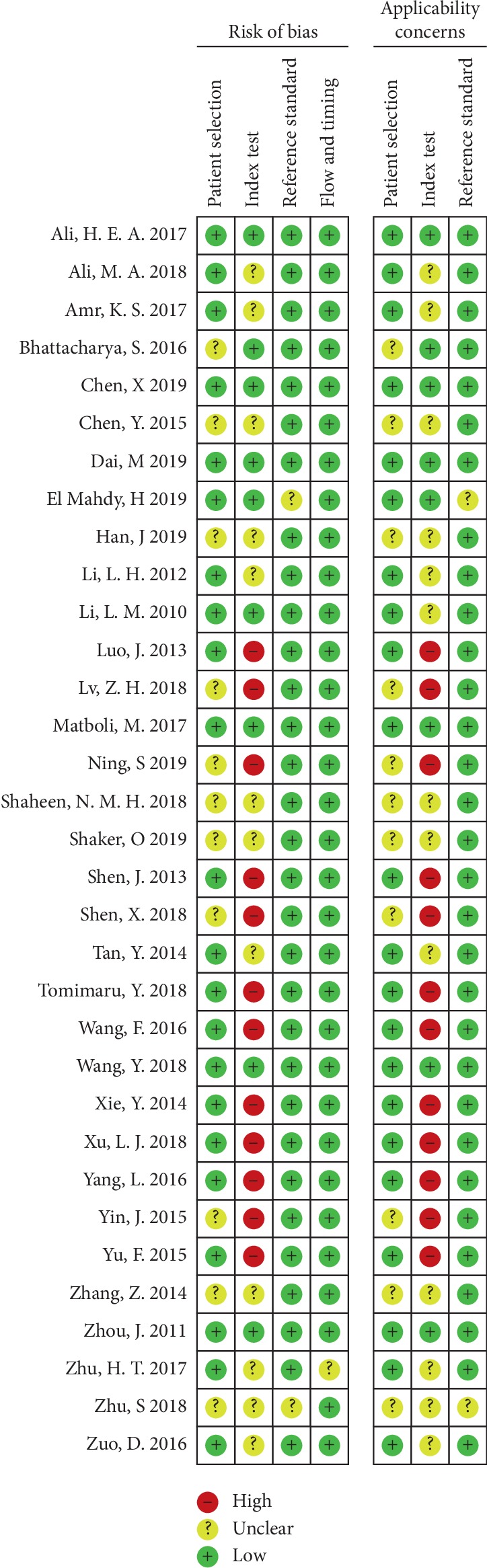
Methodological quality diagram. The overall quality assessment in the included studies based on the questions of QUADAS-2 quality assessment. The red, yellow, and green colors show the high, unclear, and low risk of bias and applicability concerns, respectively.

According to the QUADAS-2 tools, we provided an overview of the quality assessment for these studies. In the Index Test aspect, there existed a high risk of bias and applicability concern due to presetting the threshold.  Figure 2 shows the details of the quality assessment form.

### 3.4. Comprehensive Analysis

**Figure 3:**
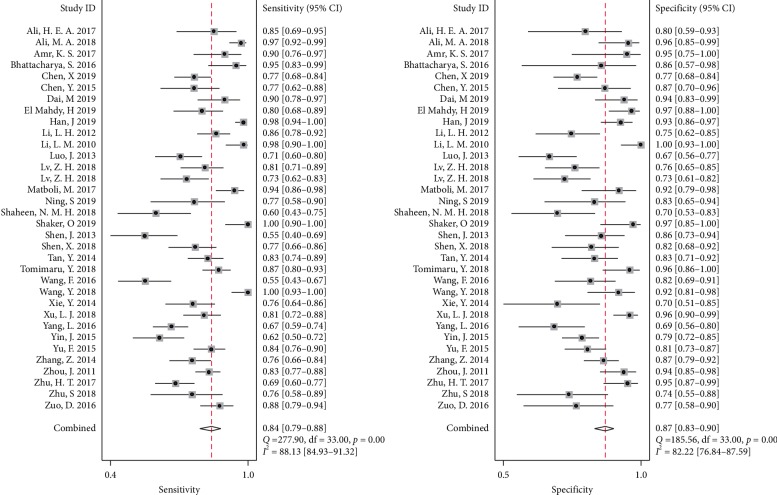
The forest plot of miRNAs for overall diagnostic efficacy in HCC. The solid squares represent the point estimates for the sensitivity and specificity of each study. Error bars indicate 95% confidence interval (CI).

**Figure 4:**
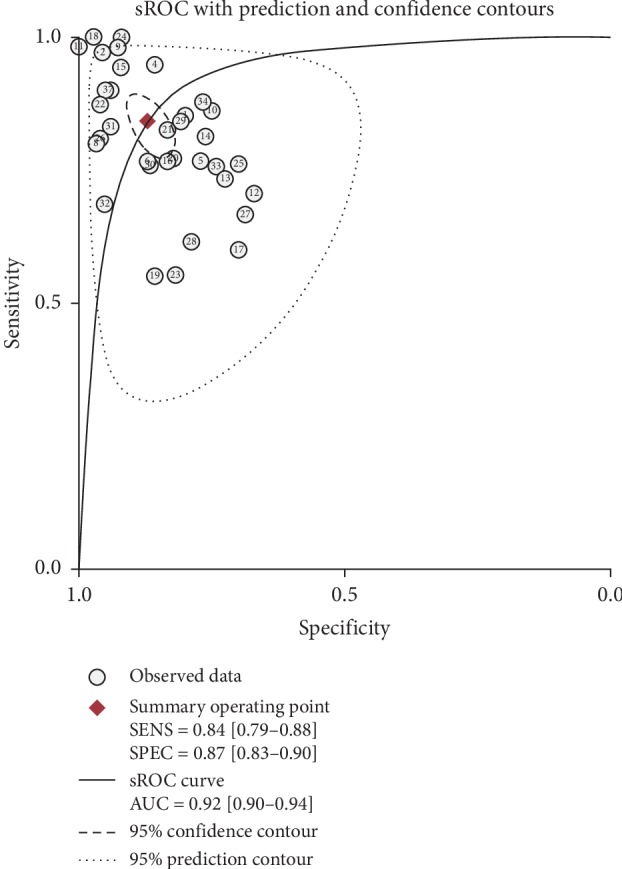
The summary receiver operator characteristic (sROC) curve of miRNAs for the diagnosis of HCC. The numerical value in each circle represents the number of the included studies in the meta-analysis. And the regression sROC curve indicates overall diagnostic accuracy.

The threshold effect was evaluated by the Spearman correlation coefficient (−0.537), with the *P* value of 0.001, showing that the threshold effect existed. Then we analyzed the pooled sensitivity and specificity of miRNAs, with 0.84 (95% CI, 0.79–0.88) sensitivity, 0.87 (95% CI, 0.83–0.90) specificity (Figure 3), and 0.92 (95% CI, 0.90–0.94) AUC in sROC curve (Figure 4). Heterogeneity was found, with *I*^2^ of 88.13% in sensitivity and 82.22% in specificity, indicating that the heterogeneity was significant. Subsequently, subgroup analyses were conducted to explore the possible sources of heterogeneity.

### 3.5. Subgroup Analyses

**Figure 5:**
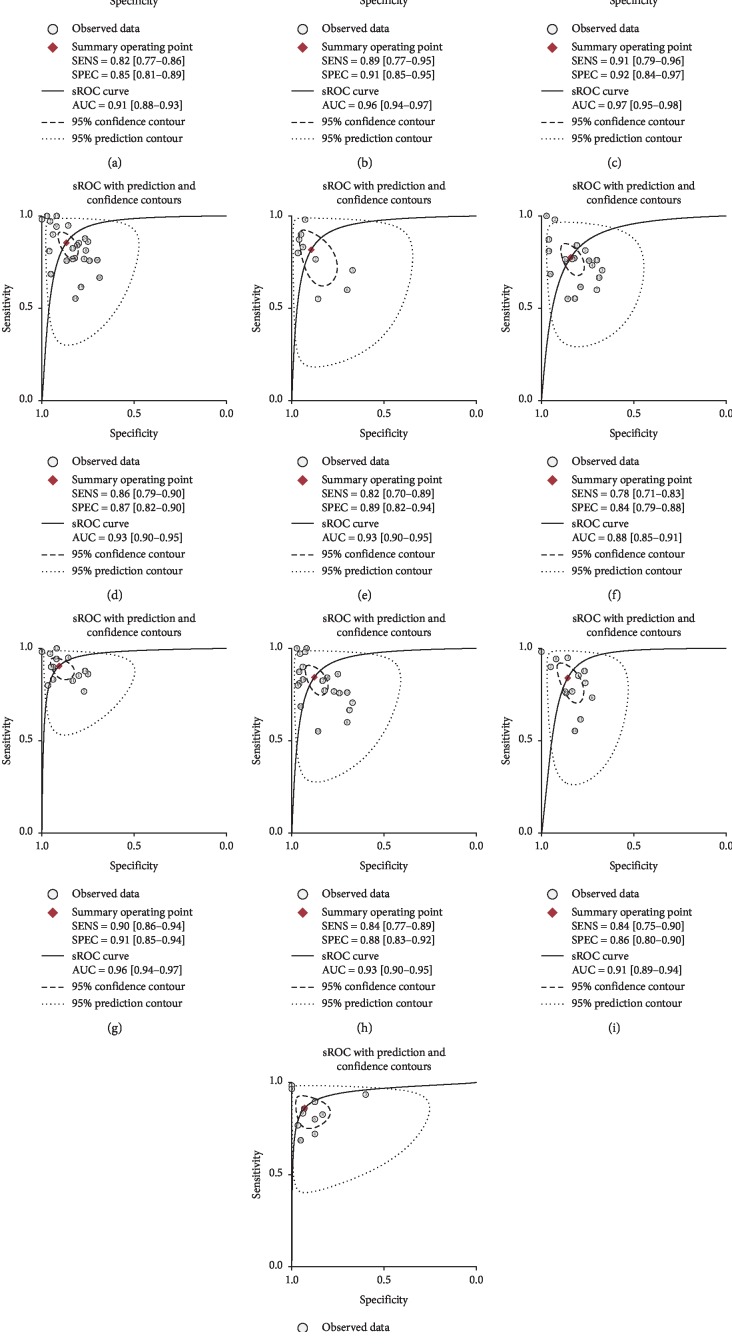
sROC curve for subgroup analyses. sROC curve describes the diagnostic performance of miRNAs in discriminating HCC in (a) China, (b) non-China, (c) Egypt, (d) serum, (e) plasma, (f) low-quality, (g) high-quality, (h) single miRNA, (i) multiple miRNAs, and (j) miRNA panel subgroups from healthy individuals, and each solid circle represents a included study in our meta-analysis.

We divided these studies into four subgroups, including sample, nation, quality, and miRNA profiling. In the nation subgroup, we divided the studies into three groups: China, non-China, and Egypt groups. We found the studies on the Egypt people had superior diagnostic efficacy, with 0.91 (95% CI, 0.79–0.96) sensitivity, 0.92 (95% CI, 0.84–0.97) specificity, 0.97 (95% CI, 0.95–0.98) AUC, 12.0 (95% CI, 5.2–27.5) positive likelihood ratio (PLR), 0.10 (95% CI, 0.04–0.24) negative likelihood ratio (NLR), and 119 (95% CI, 24–592) diagnostic odds ratio (DOR), showing miRNAs had better diagnostic ability for HCC in Egypt. The studies were divided into low- or high-quality subgroup according to the result of quality assessment. Figures 5(a)–5(j) show the diagnostic effect in China, non-China, Egypt, serum, plasma, low-quality, high-quality, single miRNA, multiple miRNAs, and miRNA panel subgroups. The diagnostic efficacy of serum- and plasma-derived miRNAs was the same, with 0.93 AUC (95% CI, 0.90–0.95). And the high-quality subgroup had 0.90 (95% CI, 0.86–0.94) sensitivity, 0.91 (95% CI, 0.85–0.94) specificity, 93 (95% CI, 40–214) DOR, and 0.96 (95% CI, 0.94–0.97) AUC, higher than low-quality subgroup. And miRNA panel had better diagnostic efficacy than single miRNA and multiple miRNAs subgroup, with sensitivity of 0.86 (95% CI, 0.79–0.91), specificity of 0.93 (95% CI, 0.85–0.97), PLR of 12.2 (95% CI, 5.7–27.0), NLR of 0.15 (95% CI, 0.10–0.23), DOR of 81 (95% CI, 28–236), and AUC of 0.95 (95% CI, 0.92–0.96).  Table 2 shows the detailed results of subgroup analyses.

### 3.6. Meta-Regression Analysis

**Figure 6:**
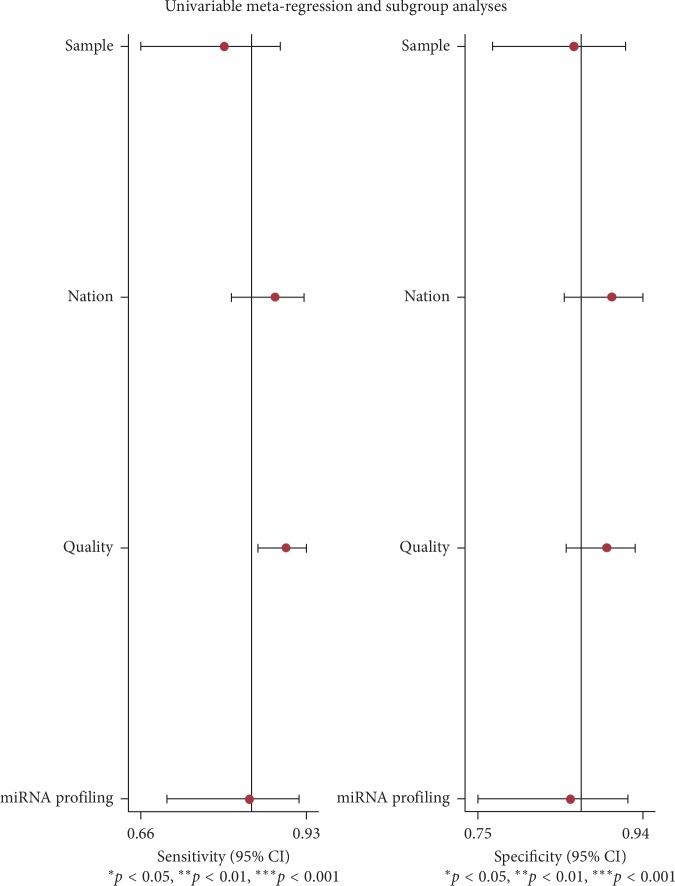
Meta-regression for subgroups. Univariate meta-regression and subgroup analyses of sensitivity and specificity. ^*∗*^, ^*∗∗*^, and ^*∗∗∗*^ show the statistically significant difference.

Meta-regression analysis was used to investigate the possible sources of heterogeneity in Meta-Disc 1.4 software. Since all of *I*^2^ in sensitivity, specificity, PLR, NLR, and DOR were more than 70% ([Supplementary-material supplementary-material-1]), we explored the source of heterogeneity. The *P* values were 0.0410, 0.9808, 0.3906, and 0.5372 in quality, sample, nation, and miRNA profiling subgroups, respectively. We also separately analyzed the impact of quality on the meta-analysis. The variable of quality had a *P* value with 0.0093, demonstrating the quality of studies was the main source of heterogeneity (Table 3). Meanwhile, we also performed meta-regression analysis using Stata 14.0 software. However, the difference in quality was not statistically significant in Figure 6.

### 3.7. Publication Bias

**Figure 7:**
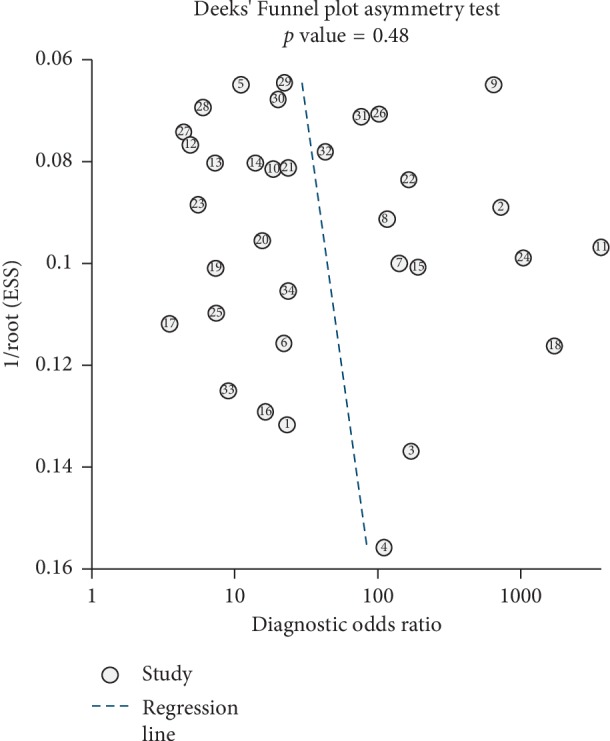
Deeks' funnel plot asymmetry test was used to perform the assessment of the publication bias.

In order to evaluate the underlying publication bias, Deeks' funnel plot was designed in Stata 14.0 software. The *P* value was 0.48, indicating the probability of publication bias was fairly small (Figure 7).

## 4. Discussion

Early HCC patients are usually asymptomatic [67], which made the diagnosis of HCC more difficult. When the HCC patients have obvious symptoms, such as liver pain, jaundice, refractory ascites, progressive weight loss, fever, cachexia, or very serious complications (hepatic encephalopathy), indicating that early HCC may progress into advanced stages [68, 69], then the treatments will be limited. The radical hepatic resection in early HCC is one of the most effective treatments [70]. However, only 30% to 40% of HCC patients can perform radical treatment at the time of diagnosis [63]. Therefore, the early diagnosis of HCC is rather important for improving the five-year survival rate of HCC patients. In recent years, miRNAs have been found to be potential biomarkers for the diagnosis of HCC [44, 63]. However, the conclusions are inconsistent. Therefore, we conducted this study to evaluate whether miRNAs can be used as diagnostic biomarkers for early HCC.

In this study, the overall sensitivity, specificity, and AUC were 0.84, 0.87, and 0.92, respectively, indicating that the overall accuracy was high using the circulating miRNAs as diagnostic biomarkers for HCC. In addition, the 6.5 PLR showed better diagnostic efficacy for distinguishing HCC patients from healthy individuals. The 0.18 NLR showed miRNAs had the probability of excluding the participants without HCC. The value of DOR (36, 95% CI: 20–64) showed high diagnostic efficacy in 34 studies. Subsequently, we analyzed the main source of heterogeneity and divided these studies into four subgroups. It was reported that the circulating miRNA concentrations might be associated with different ethnic groups [71]. Then, we found the studies in Egypt had better diagnostic accuracy than China. And Shaker et al. found the incidence of HCC in Egypt is overall increasing, from 4% in 1993 to 7.3% in 2003 [67]. Besides, the incidence rate of HCC among the cirrhosis patients in Egypt was approximately 21% [41], which might play an important role in the diagnosis of HCC. In addition, Yang, Y et al. found the miRNAs had higher diagnostic efficacy in Asians with 22 DOR than Caucasians [72]. Therefore, multiple-central studies are needed to verify our findings. Furthermore, the miRNAs were reported that they were differentially expressed in plasma and serum [73]. Then we explored the diagnostic efficacy of miRNAs in serum and plasma for HCC. Intriguingly, the serum and plasma subgroup had the same AUC and 95% CI. Like our study, Yang et al. also found the diagnostic efficacy was not statistically significant in the two sample types through a meta-analysis [72]. We speculated that serum- or plasma-derived miRNAs might have little difference on the diagnosis of HCC. However, due to the lack of consensus on whether plasma or serum is more suitable for sample detection, there exist limitations to analyzing the expression level of miRNAs in both plasma and serum [74]. Subsequently, we found the high-quality studies had better diagnostic efficacy than low-quality studies, showing the quality of studies was one of the most essential factors influencing the overall heterogeneity. And in the meta-regression, we found the difference in quality was statistically significant using Meta-disc 1.4 software, with *P* value <0.05. Besides, we also showed that miRNA panel had a higher diagnostic value than multiple miRNAs or single miRNA subgroup. Hung et al. showed that miRNA panel had 84.8% sensitivity and 50.0% specificity, better than single miRNA (miR-122 and let-7b) [75]. Zhou et al. also reported that miRNA panel (miR-122, miR-192, miR-21, miR-223, miR-26a, miR-27a, and miR-801) can distinguish early HCC from healthy individuals with 82.5% sensitivity, 83.5% specificity, and 0.888 AUC [63]. And Zhang et al. also showed that 3-miRNA panels (miR-92a-3p, miR-107, and miR-3126-5p) had better diagnostic accuracy with 0.975 AUC [76]. Ning et al. also demonstrated miRNA panel (miR-155, miR-96, and miR-99a) had 0.931 AUC, higher than single miRNA [48]. Meanwhile, Pascut et al. provided comprehensive profiling of miRNome in HCC patient blood and serum, which provided useful molecular markers for the diagnosis of HCC [77].

Our study had some advantages compared with previous studies. Firstly, we integrated multiple single miRNAs or miRNA panels into a single miRNA in a study for improving the diagnostic efficacy of miRNAs. Secondly, we evaluated the diagnostic performance of circulating miRNAs in serum or plasma for early HCC patients. Thirdly, we combined the Stata 14.0 software, Review Manager 5.3 [78], and Meta-Disc 1.4 software to perform the meta-analysis. And we also analyzed the difference of diagnosis for HCC among single miRNA, multiple miRNAs, and miRNA panel. Ultimately, our results were promising and implied that miRNAs might be potential noninvasive biomarkers for the diagnosis of early HCC.

Nevertheless, there existed several limitations to the present study. First of all, there existed a threshold effect, which might be related to the cutoff values. For example, Hea et al. set the cutoff value of miR-126 and miR-21 to 0.462 and 4.26, respectively [34]. Secondly, the subgroup classifications of HCC based on the different background, such as chronic hepatitis B, chronic hepatitis C, other types of nonviral hepatitis, and liver cirrhosis, were not conducted because some studies lack the detailed information. Furthermore, these studies lacked united internal reference in RNA quantification [79]. In the included articles, most of the studies used RNA U6 (also called snRNA U6 or U6), while some studies used miR-16, miR-39, or other miRNAs as internal reference.

In conclusion, our results have shown miRNAs in vivo can be acted as a potential diagnostic biomarker for HCC, which can promote the diagnosis of early HCC in clinical practice. Additionally, we also found miRNA panel in serum or plasma may have better diagnostic efficacy than single miRNA. In addition, more high-quality and multiple-central studies are needed to verify our findings.

## Figures and Tables

**Table 1:** Characteristics of studies.

Study	Year	Country	Specimen size		Age ± SD (years)		Male (%)		Method	Internal reference	Ref
			HCC	HC	HCC	HC	HCC	HC			
Ali, H. E. A.	2017	Egypt	34	25	na	na	76.50	72.00	qRT-PCR	miR-16	[34]
Ali, M. A.	2018	Egypt	105	45	na	na	66.70	53.30	qRT-PCR	RNA U6	[35]
Amr, K. S.	2017	Egypt	40	20	52.03 ± 1.55	50.75 ± 1.80	82.50	80.00	RT-PCR	RUN6B	[36]
Bhattacharya, S.	2016	Columbia	39	14	58.00 ± 12.00	38.00 ± 9.00	78.57	71.43	qRT-PCR	miR-39	[37]
Chen, X.	2019	China	120	118	na	na	81.67	na	qRT-PCR	miR-16	[38]
Chen, Y.	2015	China	47	31	na	na	93.62	58.06	qPCR	miR-16	[39]
Dai, M.	2019	China	50	50	48.60 ± 11.90	47.20 ± 11.70	70.00	70.00	RT-qPCR	U6	[40]
El Mahdy, H.	2019	Egypt	60	60	53.97 ± 6.15	51.67 ± 6.40	58.33	53.33	RT-qPCR	RNU6	[41]
Han, J.	2019	China	155	96	58.20 ± 10.50	51.10 ± 13.70	81.94	63.54	RT-PCR	U6 snRNA/Cel-miR-39	[42]
Li, L. H.	2012	China	86	60	54.00 ± 11.00	52.00 ± 16.00	75.25	76.67	qRT-PCR	U6 snRNA	[43]
Li, L. M.	2010	China	55	50	52.83 ± 7.85	47.72 ± 12.09	83.64	84.00	qRT-PCR	miR-168	[44]
Luo, J.	2013	China	85	85	53.60 ± 12.0	50.80 ± 13.00	82.40	81.20	qPCR	U6RNA	[45]
Lv, Z. H.	2018	China	75	75	na	na	na	na	na	RNU6	[46]
Lv, Z. H.	2018	China	75	80	52.80 ± 9.70	na	94.70	na	qRT-PCR	miR-39	[46]
Matboli, M.	2017	Egypt	70	38	na	na	74.30	84.20	qPCR	RNU-6	[47]
Ning, S.	2019	China	30	30	50.70 ± 10.56	49.80 ± 10.76	60.00	53.30	RT-qPCR	miR-16	[48]
Shaheen, N. M. H.	2018	Egypt	40	40	58.00 ± 8.60	57.50 ± 10.00	65.00	85.00	qRT-PCR	cel-miR-39	[49]
Shaker, O.	2019	Egypt	36	36	59.92 ± 7.47	56.86 ± 6.38	77.78	69.44	RT-PCR	SNORD68	[50]
Shen, J.	2013	New York	49	49	61.10 ± 11.70	61.50 ± 11.00	84.00	84.00	qRT-PCR	U6snRNA	[51]
Shen, X.	2018	China	70	45	na	na	65.71	na	qRT-PCR	U6	[52]
Tan, Y.	2014	China	103	60	52.01 ± 10.21	41.43 ± 7.76	73.80	70.00	qRT-PCR	miRNA-24	[53]
Tomimaru, Y.	2012	China	126	50	63.00 ± 10.00	62.00 ± 8.00	78.60	74.00	qRT-PCR	miR-16	[54]
Wang, F.	2016	China	76	55	na	na	86.84	na	qRT-PCR	cel-miR-39	[55]
Wang, Y.	2018	China	50	50	56.32 ± 9.71	53.92 ± 8.17	80.00	74.00	qPCR	na	[56]
Xie, Y.	2014	China	67	30	51.69 ± 10.43	37.26 ± 10.79	85.10	70.00	qRT-PCR	cel-miR-39	[57]
Xu, L. J.	2018	China	100	100	54.90 ± 13.10	54.10 ± 13.80	74.00	74.00	qRT-PCR	miR-U6	[58]
Yang, L.	2016	China	156	64	na	na	na	na	qRT-PCR	RUN6B	[59]
Yin, J.	2015	China	78	156	56.30 ± 6.70	55.80 ± 7.10	37.18	34.62	qRT-PCR	U6SnRNA	[60]
Yu, F.	2015	China	120	120	58.00 ± 10.40	50.00 ± 9.50	62.5	54.17	qRT-PCR	miR-16	[61]
Zhang, Z.	2014	China	95	127	54.21 ± 6.95	52.58 ± 6.98	63.16	55.91	qRT-PCR	U6	[62]
Zhou, J.	2011	China	196	66	53.00 ± 12.00	45.00 ± 12.00	85.00	65.00	qRT-PCR	miR-1228	[63]
Zhu, H. T.	2017	China	121	62	na	na	84.30	58.06	qPCR	na	[64]
Zhu, S.	2018	China	33	31	54.00 ± 11.00	51.00 ± 9.00	81.82	96.77	RT-qPCR	U6 snRNA	[65]
Zuo, D.	2016	China	90	30	54.70 ± 9.80	51.80 ± 20.20	75.56	36.67	RT-PCR	RNA U6	[66]

HCC: hepatocellular carcinoma; HC: healthy control; Ref: reference; na: not available.

**Table 2:** The results of subgroup analysis.

Subgroup	Sensitivity (95% CI)	Specificity (95% CI)	PLR (95% CI)	NLR (95% CI)	DOR (95% CI)	AUC (95% CI)
*Sample*						
Serum	0.86 (0.79, 0.90)	0.87 (0.82, 0.90)	6.5 (4.6, 9.1)	0.17 (0.11, 0.25)	39 (19, 78)	0.93 (0.90, 0.95)
Plasma	0.82 (0.70, 0.89)	0.89 (0.82, 0.94)	7.8 (4.0, 14.9)	0.20 (0.12, 0.36)	38 (12, 117)	0.93 (0.90, 0.95)

*Nation*						
China	0.82 (0.77, 0.86)	0.85 (0.81, 0.89)	5.7 (4.1, 7.8)	0.21 (0.16, 0.28)	27 (15, 48)	0.91 (0.88, 0.93)
Non-China	0.89 (0.77, 0.95)	0.91 (0.85, 0.95)	10.3 (5.3, 20.1)	0.12 (0.05, 0.27)	89 (22, 236)	0.96 (0.94, 0.97)
Egypt	0.91 (0.79, 0.96)	0.92 (0.84, 0.97)	12.0 (5.2, 27.5)	0.10 (0.04, 0.24)	119 (24, 592)	0.97 (0.95, 0.98)

*Quality*						
Low-quality	0.78 (0.71, 0.83)	0.84 (0.79, 0.88)	4.9 (3.5, 6.9)	0.27 (0.19, 0.37)	18 (10, 34)	0.88 (0.85, 0.91)
High-quality	0.90 (0.86, 0.94)	0.91 (0.85, 0.94)	9.7 (6.0, 15.9)	0.11 (0.07, 0.16)	93 (40, 214)	0.96 (0.94, 0.97)

*miRNA profiling*						
Single miRNA	0.84 (0.77, 0.89)	0.88 (0.83, 0.92)	6.9 (4.6, 10.4)	0.18 (0.12, 0.27)	39 (18, 83)	0.93 (0.90, 0.95)
Multiple miRNAs	0.84 (0.75, 0.90)	0.86 (0.80, 0.90)	5.9 (3.9, 8.9)	0.19 (0.11, 0.31)	32 (13, 75)	0.91 (0.89, 0.94)
miRNA panel	0.86 (0.79, 0.91)	0.93 (0.85, 0.97)	12.2 (5.7, 27.0)	0.15 (0.10, 0.23)	81 (28, 236)	0.95 (0.92, 0.96)

PLR: positive likelihood ratio; NLR: negative likelihood ratio; DOR: diagnostic odds ratio; AUC: area under the curve.

**Table 3:** The meta-regression of covariates.

Variable	Coefficient	Standard error	*P* value	RDOR (95% CI)
*Model 1: the variables are quality, sample, nation, and miRNA profiling*				
Cte.	2.358	1.1800	0.0555	—
*S*	0.123	0.2919	0.6768	—
Quality	0.641	0.2993	0.0410	1.90 (1.03, 3.51)
Sample	−0.012	0.5037	0.9808	0.99 (0.35, 2.77)
Nation	0.320	0.3668	0.3906	1.38 (0.65, 2.92)
miRNA profiling	−0.335	0.5356	0.5372	0.72 (0.24, 2.14)

*Model 2: the variables are quality, nation, and miRNA profiling*				
Cte.	2.336	0.9455	0.0196	—
*S*	0.12	0.2838	0.6746	—
Quality	0.642	0.2872	0.0334	1.90 (1.06, 3.42)
Nation	0.316	0.3519	0.3762	1.37 (0.67, 2.82)
miRNA profiling	−0.332	0.5243	0.5316	0.72 (0.25, 2.10)

*Model 3: the variables are quality and nation*				
Cte.	1.89	0.627	0.0052	—
*S*	0.11	0.2798	0.6981	—
Quality	0.643	0.2830	0.0304	1.90 (1.07, 3.39)
Nation	0.301	0.3465	0.3921	1.35 (0.67, 2.74)

*Model 4: the variable is quality*				
Cte.	2.155	0.5404	0.0004	—
*S*	0.09	0.2765	0.7473	—
Quality	0.728	0.2626	0.0093	2.07 (1.21, 3.54)

RDOR: relative diagnostic odds ratios. *P* value <0.05 showing the significant difference.

## Data Availability

The data used to support the findings of this study are included within the article.

## Supplementary Materials

Supplementary Materials Table S1: the diagnostic efficacy of miRNAs in the included studies (Supplementary Material 1). Figure S2: the pooled (a) sensitivity, (b) specificity, (c) PLR, (d) NLR, and (e) DOR were obtained using Meta-disc 1.4 software (Supplementary Material 2).
