# Supplementary figures and images for: The Diagnostic Value of MicroRNAs as a Biomarker for Hepatocellular Carcinoma: A Meta-Analysis
Source: Biomed Res Int. 2019 Nov 29;2019:5179048. doi: 10.1155/2019/5179048 (PMC6907051; doi:10.1155/2019/5179048)

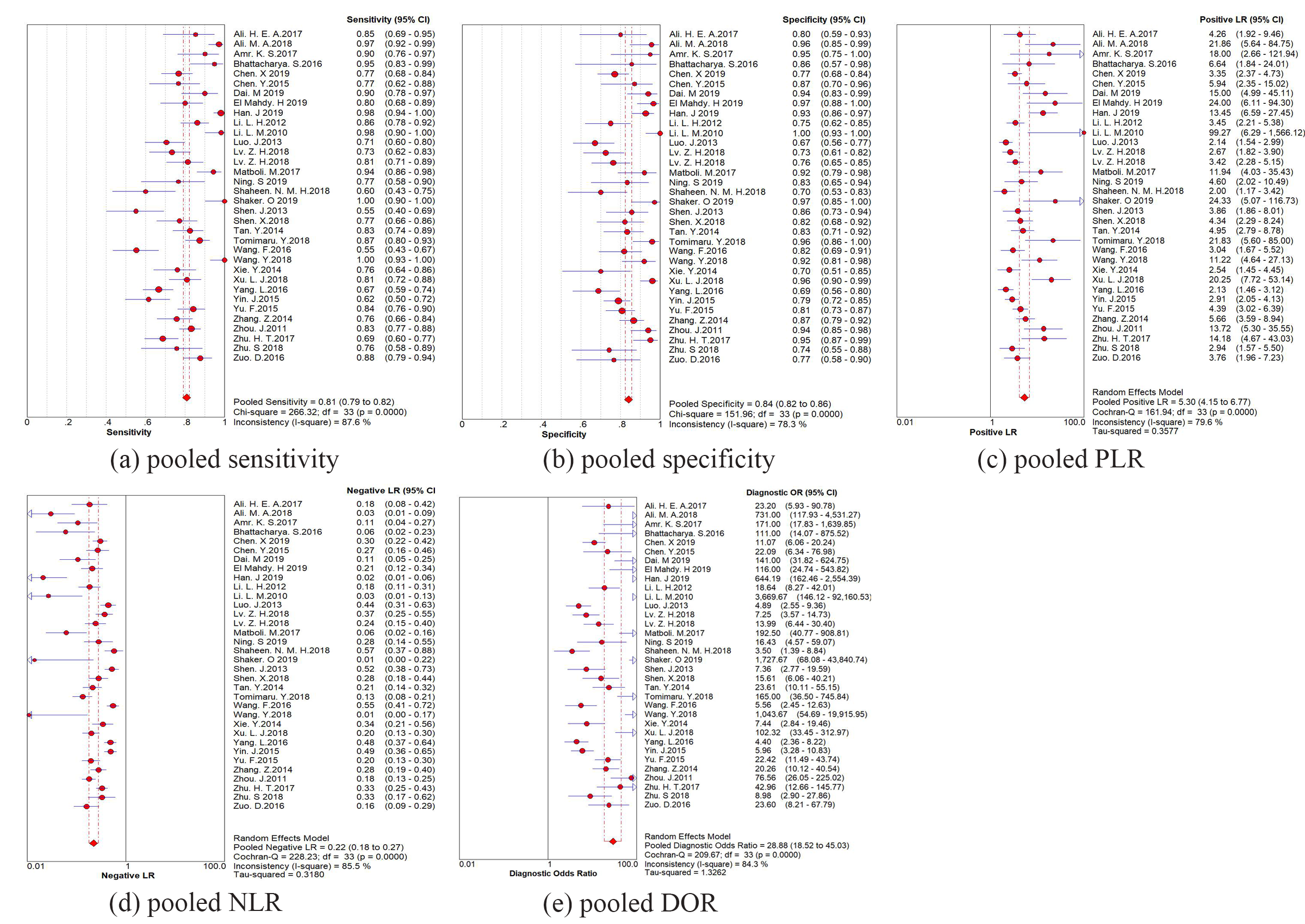

Supplement: Supplementary Materials — Table S1: the diagnostic efficacy of miRNAs in the included studies (Supplementary Material 1). Figure S2: the pooled (a) sensitivity, (b) specificity, (c) PLR, (d) NLR, and (e) DOR were obtained using Meta-disc 1.4 software (Supplementary Material 2). [file 5179048.f1.zip › 5179048.f1/Supplementary Material 2 Figure S2 The pooled (a) sensitivity, (b) specificity, (c) PLR, (d) NLR, (e) DOR.tif]
